# Feasibility of group-based acceptance and commitment therapy for adolescents (AHEAD) with multiple functional somatic syndromes: a pilot study

**DOI:** 10.1186/s12888-020-02862-z

**Published:** 2020-09-21

**Authors:** Karen Hansen Kallesøe, Andreas Schröder, Rikard K. Wicksell, Tua Preuss, Jens Søndergaard Jensen, Charlotte Ulrikka Rask

**Affiliations:** 1 Research Clinic for Functional Disorders and Psychosomatics, Aarhus University Hospital, Noerrebrogade 44, building 2C, 8000 Aarhus C, Denmark; 2 Department of Clinical Medicine, Aarhus University, Aarhus, Denmark; 3 Department of Clinical Neuroscience, Karolinska Institutet, Stockholm, Sweden; 4 Department of Child and Adolescent Psychiatry, Psychiatry, Aarhus University Hospital, Aarhus, Denmark

**Keywords:** Acceptance and commitment therapy (ACT), Adolescents, Functional somatic syndromes, Group-therapy, Psychotherapy

## Abstract

**Background:**

Recurrent and impairing functional somatic syndromes (FSS) are common in adolescents. Despite a high need for care, empirically supported treatments are lacking for youth. The aim of this uncontrolled pilot study was to assess feasibility and treatment potential of a new intervention with group-based Acceptance and Commitment Therapy (ACT) in a generic treatment approach for adolescents with multiple FSS.

**Methods:**

Twenty-one patients received ‘ACT for Health in Adolescents’ (AHEAD) (30 h), specifically developed for adolescents (aged 15–19 years) with moderate to severe FSS. Close relatives attended an information meeting to facilitate support of the patients throughout treatment. Treatment satisfaction was evaluated by means of self-report and relatives’ impressions. Self-reported physical health at 3 months follow-up (FU) after end of treatment was the primary outcome whereas secondary outcomes included symptom burden, limitation due to symptoms, illness worry, emotional distress and physical and emotional symptoms. Treatment targets were assessed by measures on illness behaviour, illness perception and psychological inflexibility.

**Results:**

Nineteen patients (90.5%) completed the treatment with a high overall attendance rate of 93%. All would recommend the treatment to a friend with similar problems. Close relatives rated it valuable to participate in an information meeting. Patients’ physical health improved significantly from assessment to FU with a clinically relevant mean change of 8.9 points (95% CI [5.4; 12.4]; SRM 0.91 [0.26;1.57]). Improvement was also seen on all secondary outcome measures, from assessment to FU. Maladaptive illness behaviours and perceptions as well as psychological inflexibility showed a significant decline from assessment to FU.

**Conclusion:**

AHEAD was feasible and potentially efficacious and warrants testing in a larger clinical trial.

**Trial registration:**

Clinical Trials gov NCT04464447, registration date July 9th, 2020. Retrospectively registered.

## Background

An increasing number of adolescents report daily unspecific physical symptoms [1]. While the symptoms are self-limiting in most cases, approximately 5–10% report persistent symptoms, and may receive diagnoses of functional somatic syndromes (FSS) [2]. FSS collectively refer to a range of syndromes (e.g. chronic fatigue syndrome, idiopathic pain syndrome, juvenile fibromyalgia and functional gastrointestinal disorders (FGID)), characterized by a pattern of impairing physical symptoms for which no well-defined physical disease can be identified. Often comorbid anxiety and depression exist [3–5].

The aetiology of FSS is assumed complex with interacting biological, psychological and environmental factors [2, 6–8]. Suffering from FSS during adolescence can have serious consequences in terms of school absence, social withdrawal and reduced quality of life [2]. Frequently, symptoms sustain into adulthood, carrying a significant risk of long-term impairment, reduced probability of obtaining a higher education and a high use of health care costs [9–13]. Known maintaining factors include negative illness perceptions (e.g. single cause attribution of symptom, no sense of control and long expectancy of symptoms) [14–16] and maladaptive illness behaviours (e.g. avoidance, control and ‘all-or-nothing’ behaviour) [17]. Furthermore, parental overprotective behaviour can increase distress and disability in the young [18–21], and better outcome is suggested when parents support a shift for more accommodative coping styles [22, 23].

Recent reviews show that psychological interventions, particularly based on cognitive behavioural therapy (CBT) principles, are effective for FSS in children and adolescents in reducing symptom load, disability and school absence [24, 25]. Acceptance and commitment therapy (ACT) is a newer development within CBT that has shown promising results in children and adolescents with chronic pain [26–28] and in adults with various FSS [28–31]. The overall aim of ACT is to change how the individual reacts to unwanted inner experiences by broadening the behavioural repertoire, to facilitate more adaptive strategies. This is done within the contextual framework of increasing ‘psychological flexibility’, i.e. the ability to take actions in accordance with own life values also in the presence of unpleasant physical and emotional experiences [32]. Psychological flexibility is increased by the training of six core processes (i.e. acceptance, cognitive defusion, being present in the moment, self as context, values and committed action). Hence, the overall focus in ACT is to improve functioning by a shift in focus from alleviation of symptoms to acceptance of inner experiences and commitment to engaging in values-based behaviours. Hereby ACT targets inefficient coping strategies such as symptom avoidance and control behaviour [33–35]. Furthermore, as complete symptom elimination may not be realistic in youth with multiple FSS, the strong focus on values may serve as a motivational factor for behavioural change [36].

In adults with multiple FSS, a generic group-based treatment approach has been found effective regardless of main symptom [37]. The clinical rationale for a generic treatment approach is further supported by the empirically proved overlap in symptomatology between patients with various FSS [38, 39]. Despite high co-occurrence of symptoms from different organ systems is also described in youth [40, 41], research has so far focused on treatment of single syndrome FSS based on sub-specialties in the paediatric setting [24]. Hitherto one pilot study presents data on a generic treatment across symptom profiles in an adolescent population [42]. Further research on generic treatment options has been suggested to diminish the risk of fragmented care in specialized clinics [24, 43–45]. Therefore, in this pilot study we evaluated a new generic ACT-based group treatment for adolescents with multiple FSS, conceptualized under the unifying diagnostic category of multi-organ Bodily Distress Syndrome (BDS) [46]. The use of BDS as a diagnostic conceptualization for functional disorders is in line with the recommendations by Burton et al. [47].

Our main aim of the present study was to explore the feasibility of a group-based ACT intervention for adolescents with multiple FSS, i.e. treatment adherence, satisfaction and overall experience with the treatment reported by patients and close relatives. Furthermore, we wanted to explore preliminary efficacy testing and changes in suggested treatment targets, i.e. illness perception, illness behaviour and psychological inflexibility pertaining to the total intervention (i.e. assessment, psychoeducation, and group-based treatment).

## Method

### Design and setting

This was an open pilot trial. Enrolment started in May 2013, and data collection was finalised in April 2015. Young patients were referred from general practitioners, hospital departments or medical specialists to a specialized university hospital service, for assessment and treatment of debilitating FSS.

### Trial registration

Before commencement, the trial was registered at the Danish Data Protection Agency (no. 1–16–02-261-14) whereas the National Committee on Health Research Ethics, Denmark waived registration (contact no. 93/2013) due to the feasibility nature of the study. The trial was retrospectively registered at Clinical Trials Gov NCT04464447.

### Eligibility criteria

Eligibility criteria were age 15–19 years, multi-organ BDS, i.e. at least three functional somatic symptoms from at least three symptom groups, moderate to severe impairment in daily life and symptom duration of minimum 12 months (see Table 1) [46, 48].

**Table 1:** In- and exclusion criteria

Inclusion criteria	
1. Bodily Distress Syndrome, multi-organ type of at least 12 months’ duration.	
2. 15–19 years old at referral.	
3. Raised since early childhood in Denmark or born by Danish parents. Understand, speak and read Danish.	
4. Moderate or severe impairment.	
Exclusion criteria	
1. Acute psychiatric disorder demanding other treatment, or if the patient is suicidal.	
2. A lifetime diagnosis of psychosis, mania or depression with psychotic symptoms (ICD-10: F20–29, F30–31, F32.2, F33.3), serious cognitive deficits or developmental disorders such as mental retardation and autism (ICD-10: F70, F84)	
3. Substance abuse of e.g. narcotics, alcohol or medication.	
4. Pregnancy at the time of inclusion.	
5. Not able to participate in group-based treatment, e.g. patients with severe ADHD (ICD-10: F90), severe social phobia (ICD-10: F40.1) or conduct disorder (ICD-10: F91).	

### Procedures

#### Clinical assessment

Referrals were screened for eligibility by a physician (CUR). Eligible patients were invited for a standardized clinical psychiatric and somatic assessment which was performed by physicians trained in child and adolescent psychiatry (KHK and CUR). It consisted of 1) a systematic medical and psychosocial history taking (see Additional file 1), 2) a semi-structured diagnostic interview with Schedules for Clinical Assessment in Neuropsychiatry (SCAN) which screens for general psychopathology (e.g. depression and anxiety) and contains a detailed section with evaluation of 76 functional somatic symptoms [49] and 3) a clinical/neurological examination followed by feedback on the result of the assessment with a short psychoeducation regarding the BDS diagnosis (see below).

Impairment was rated by the physician as part of the SCAN interview based on reported degree of symptom related distress, school absence and social withdrawal from friends and leisure activities. The rating could be either mild, moderate, or severe (e.g. severe would correspond to a high degree of school absence or dropped out of school, social isolation with withdrawal from friends and cessation of previous leisure activities).

Participation in the study was voluntary. Patients and their parents (for patients younger than 18 years) were informed about the feasibility nature of the study. Patients were included after informed consent.

#### Psychoeducation

The bio-psycho-social model including the potential role of central sensitization and peripherally increased bodily stress response was used in an overall explanation of the development and maintenance of BDS [50–52]. This served as a framework for an individualized case formulation based on the patients’ history with identification of potential maladaptive coping strategies.

Approximately 2 weeks after clinical assessment, the patient and his/her parents attended a psychiatric consultation of 1½ hours, focusing on further psychoeducation and general advice on health promoting strategies regarding sleep, eating habits, physical and social activities and engagement in positive activities (see Additional file 2). Potential present life stressors (e.g. family conflicts, bullying, economic difficulties in the family) were evaluated to clarify areas where the patient or family might need additional help from other sectors. Maladaptive illness perceptions and behaviours, such as avoidance, control and ‘all-or-nothing’ behaviour, were introduced in general terms and addressed more specifically based on the case formulation if relevant. Finally, the patient chose two focus areas to work on until group start, e.g. change of eating habits or starting graded exercise.

#### Group-based ACT treatment

**Fig. 1:**
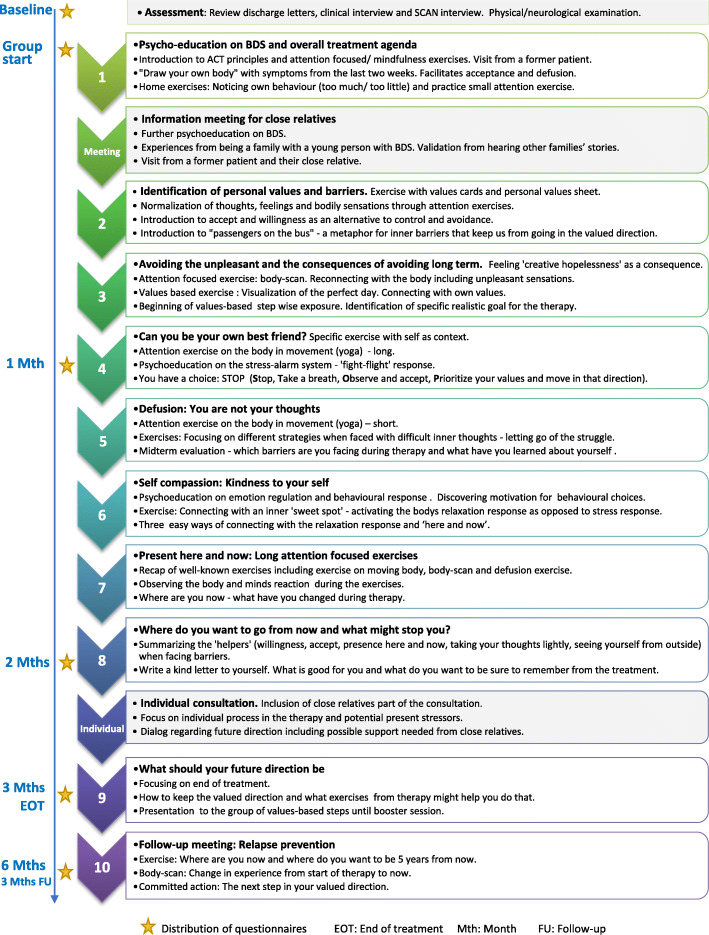
Timeline and final outline of group-based treatment

The treatment: ‘ACT for Health in Adolescents’ (AHEAD) was an ACT-based group intervention consisting of 9 modules (27 h in total) delivered over a period of 3 months, with a follow-up meeting (3 h) 3 months after the last module (Fig. 1). Six to eight patients were included in each treatment group. An information meeting (3 h) for close relatives selected by the individual patient (i.e. primarily parents but could also include adolescent siblings or a boyfriend) was held at the beginning of the group treatment, and the adolescent and the parents were invited to a 1½-hour individual consultation after the 8th module. Two therapists who also developed the treatment manual for AHEAD (CUR and TP), conjointly performed the treatment. The main therapist (a child and adolescent psychiatrist (CUR)) had classical CBT training and additional training in ACT and the co-therapist (a psychologist (TP)) had MBSR training and additional training in ACT outside the trial and both therapists had extensive knowledge of functional disorders.

The following contains a description of the final content of the treatment (see overview in Fig. 1) after minor ad hoc modifications as described in the result section.

Every treatment module was manualized. The overall aim was to support the adolescents to shift behaviour from symptom-related control and avoidance to values-based actions also when aversive symptoms are present i.e. increase psychological flexibility.

Thus, psychological inflexibility [32] was targeted by training the patients’ skills to *open up to unwanted negative experiences* (i.e. acceptance and defusion), *being present in the moment*, and *acting in accordance with life values* [53]. These skills were trained in a range of exercises during the treatment modules and supplemented by home assignments between each module.

In short, life values can be defined as freely chosen activities or constructs that give our life meaning and direction [54]. Some of the adolescents reported that they had never identified their life values and others described having lost contact with them. It was therefore a process throughout treatment to identify or re-connect with values and to take small steps in valued directions. From module 3 and onwards, each module contained a session focusing on individual graded values-based exposure i.e. identifying behavioural steps and how to take them.

*Opening up* was addressed using metaphors and exercises aimed at improving the ability to allow for the unpleasant bodily sensations without taking actions to avoid it (i.e. acceptance), in the service of remaining present in the moment. This also includes the capability of observing your thoughts, and seeing them as merely thoughts, rather than acting on them as if they represent the truth (i.e. defusion).

Similarly, *being present in the moment* is a behavioural skill that implies noticing and accepting the potentially intrusive thoughts and feelings. Thus, the treatment consisted of exercises to be conducted in session as well as between sessions, to improve the ability to redirect attention towards the present moment.

In ACT, metaphors are often used to facilitate communication regarding e.g. exposure and acceptance to previously avoided situations and reactions that may appear counter intuitive. For example, “monsters on the bus” is a metaphor used to conceptualize the negative thoughts and sensations that may occur and influence the behaviours in challenging situations when the patient is taking actions in a valued direction. The monsters represent thoughts such as “you have too many symptoms”, “you are not strong enough” that function to avoid risks. The patient is driving his/her bus through life, and choose the direction (i.e. life values) but also have to decide whether or not to listen to the passengers (acceptance) when doing so may lead away from the valued direction.

Throughout treatment, the term “helpers” was used to facilitate the adolescents’ motivation for training the new adaptive strategies described above.

The information meeting for close relatives contained three main elements: 1. Further psychoeducation on BDS, 2. Exchange of experiences regarding being a family with or close relative to an adolescent with BDS and 3. Information on treatment content, including ACT as a treatment approach, and the need for the relatives’ and families’ support during treatment.

A visit of a former patient was included in module 1 and a visit of a former patient and a parent were included at the information meeting for close relatives to inform the new participants about the patient perspective on the treatment process and create hope and motivation for change. This specific content was introduced in pilot-group 3 and was not included in pilot-group 1 and 2.

### Measures

Self-reported questionnaires were distributed before assessment, before the psychiatric consultation, before group therapy, after 4th and 8th module of therapy (1 and 2 months after treatment start, respectively), after 9th module (i.e. end of treatment (EOT) 3 months after treatment start) and after 10th module, i.e. 3-month follow-up (FU). The latter was defined as the primary endpoint.

As part of the feasibility evaluation, we examined the utility of different questionnaires to assess the defined treatment targets as well as the overall respondent burden. This meant that not all questionnaires were answered by all patients at all time-points (see Additional file 3 for details).

#### Measures of treatment satisfaction

*Patients’ satisfaction* with treatment was measured by 17 items from a modified version of The Experience of Service Questionnaire (ESQ) [55] with additional questions regarding specific treatment elements and open-end questions. The questionnaire was distributed at the follow-up meeting.

*Close relatives’ satisfaction* with the information meeting was evaluated directly after the meeting by questions concerning specific meeting content e.g. “Is the meeting content relevant” (regarding: 1. what is BDS, 2. treatment principles, 3. being a family to an adolescent with BDS) and “Are the incorporated exercises in the meeting meaningful” (regarding: 1. Presentation exercise, 2. Attention focused exercise, 3. Values-based exercise) (see Additional file 4). Also, there was a possibility of adding free text comments.

#### Primary outcome measure

The primary outcome was the aggregate score ‘physical health’ derived from The 36-Item Short Form Health Survey (SF-36) subscales PF (physical functioning), BP (bodily pain) and VT (vitality) which all have shown to be valid outcome measures in adult patients with severe FSS [56]. Thus, these three subscales have in adults shown to be sensitive to change in key areas affected in FSS and was therefore chosen as a primary outcome [57–59]. The aggregate score has previously been used in an RCT with group-based CBT for adults with multiple FSS, i.e. multi-organ BDS [37]. Scores range from 15 to 65 with higher scores indicating better self-perceived physical health. A change of 4 and above is regarded as a clinically relevant change [60–62]. Gender and age-specific Danish norm data are available from age 16 and up [63].

#### Secondary outcome measures

*Symptom severity* was measured by the BDS Checklist [64, 65] (25 items, 5-point scale), which evaluates symptoms from four symptom groups, i.e. musculoskeletal, gastro-intestinal, cardio-pulmonary and general symptoms. The sum score ranges from 0 to 100 with higher scores indicating higher symptom load. The questionnaire has shown acceptable validity [64]. A recent German validation study with inclusion of adolescents showed excellent psychometric properties and a sufficiently high internal consistency of all four subscales and the total score (all α > 0.83) [65].

*Symptom impact* was evaluated with the limitation index (LI). LI is a modified version of the Pain Interference Index (PII) (6 items, 7-point scale) [66], a validated questionnaire for children and adolescents measuring the impact of pain in performing everyday activities and impact on mood and sleep. The modification from PII to LI concerns a change in wording from ‘pain’ to ‘symptoms’. The sum score ranges from 0 to 36 with higher scores indicating a higher degree of limitation. The questionnaire has shown a high level of internal consistency (α = 0.86) in a sample of children and adolescents with chronic pain [66].

*Illness worry* was measured by Whiteley-7 [67] (7 items, 5-point scale), a subscale of the Whiteley Index. An overall mean item score ranges from 0 to 4 with higher scores indicating more severe symptoms of illness worry. Whiteley-7 has shown acceptable psychometric properties in primary care [68], and good sensitivity and specificity in screening for DSM-IV hypochondriasis [69].

*Emotional distress* was measured by SCL-8, a subscale from Symptom Checklist Revised-90 [70–73] (8 items in total, 5-point scale). An overall mean item score ranges from 0 to 4 with higher scores indicating a higher degree of emotional distress.

*The overall impression of change* was measured with Patient Global Impression of Change (PGIC) [74] (1 item, 7-point scale). Answers range from “no change (or condition has gotten worse)” to “a great deal better and a considerable improvement that has made all the difference”.

#### Measures of treatment targets

*Illness perception* was measured by the Brief Illness Perceptions Questionnaire (BIPQ) [75] (8 items, 0 to 10-point scale and an additional item regarding cause of symptoms). It evaluates the perception of five core components (identity, cause, timeline, consequences, and cure-control) which together form the perception of illness. Score ranges from 0 to 80 with higher score reflecting a more threatening view of the illness. A review and meta-analysis of the BIPQ has shown good psychometric properties across a range of populations and age-groups and has demonstrated sensitivity to change after intervention in randomised trials [76].

*Illness-related behaviour* was measured by the Behavioural Responses to Illness Questionnaire (BRIQ) [77] (13 item, 5-point scale). The questionnaire measures two dimensions of illness behaviour; (1) all-or-nothing behaviour (score range 6–30) and (2) limiting behaviour (excessive rest) (score range 7–35) with higher scores indicating a higher degree of maladaptive behaviour. Cronbach’s alphas were 0.81 (all-or-nothing behaviour) and 0.89 (limiting behaviour) in adult patients post infection and results suggested it to be a valid and reliable measure to predict the development of a functional disorder after acute infection [77].

*Psychological inflexibility* (two components, i.e. cognitive fusion and experiential avoidance) was measured by the brief version of Avoidance and Fusion Questionnaire in Youth (AFQ-Y8) [78] (8 items, 5-point scale). The total score ranges from 0 to 32 with higher scores reflecting a higher degree of avoidance and fusion. Recent studies show that AFQ-Y8 is a reliable and valid measure of these elements of psychological inflexibility in children and adolescents [79, 80].

### Statistical analysis

Data on feasibility (adherence, follow-up rates, and patient/parent satisfaction) were described by percentages. Data on primary and secondary outcomes as well as treatment targets were analysed using an unadjusted mixed model (one model for each of the above-mentioned outcome measures) with time as the only (fixed effect) covariate and a random intercept. Using this model, we calculated the mean change from assessment to follow up (FU) as well as the mean change from treatment start to FU. All available data from all participants (including two patients who discontinued participation) were included in the analysis, and the models were checked by graphical inspection of the distribution of the residuals and random intercepts. Analyses were performed using Stata version 15.1 for Windows.

## Results

### Patient characteristics

**Fig. 2:**
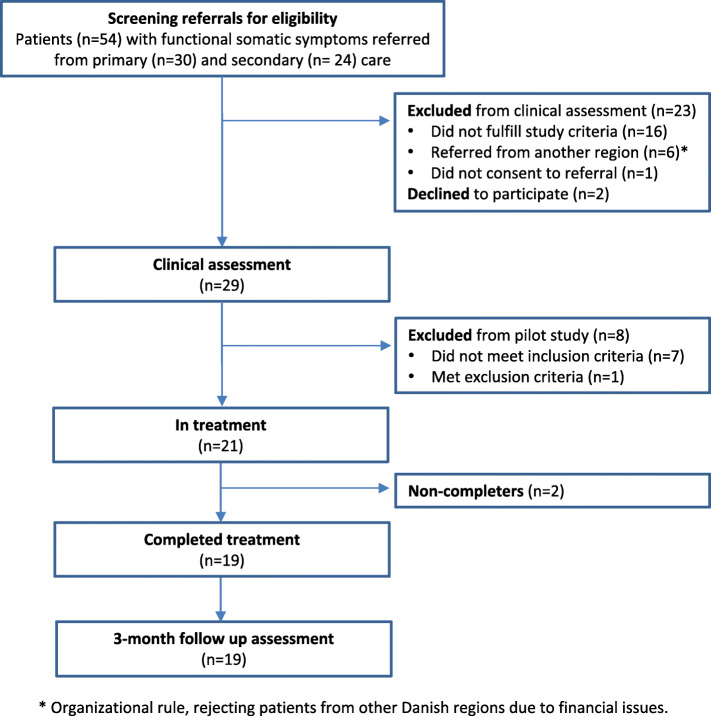
Participants’ flow through the study

A total of 54 consecutively referred adolescents were screened for eligibility; 16 did not fulfil study criteria, 6 were referred from another region (organizational rule, rejecting patients from other specific regions) and 1 did not consent to referral (Fig. 2). Thus, 29 participated in clinical assessment where 21 patients met inclusion criteria and were included. All participants were girls. Four patients who at referral had just turned 20 years were seen in the adolescent unit to increase inclusion rates in the pilot study. The mean age at inclusion was 18.5 (range 16.0–20.5) and mean duration of symptoms was 4.25 years (range 1.4–13.0) (Table 2).

**Table 2:** Patient characteristics (*N* = 21)

	Mean (range) or n (%)
Gender (female)	21 (100%)
Age, years^a^	18.5 (16.0–20.5)
Illness duration, years^a^	4.25 (1.4–13.0)
Impairment:	
Moderate	6 (28.6%)
Severe	14 (66.7%)
Family status:	
Parents divorced	12 (57.1%)
Comorbidity (medical)^b^:	8 (38.1%)
• Migraine	3 (14.3%)
• Asthma	2 (9.5%)
• Allergic rhinitis	4 (19.0%)
• Atopical dermatitis	2 (9.5%)
• Gallstone	1 (4.8%)
Comorbidity (psychiatric)^b^:	7 (33.3%)
• Hypochondriasis	3 (14.3%)
• Social phobia	2 (9.5%)
• Specific (isolated) phobia	1 (4.8%)
• Major depressive disorder, single episode, mild	1 (4.8%)
• Specific reading disorder	1 (4.8%)
Syndrome diagnoses^c^:	
Tension-type headache	21 (100%)
Non-cardiac chest pain	18 (85.7%)
Chronic fatigue syndrome (CFS)	13 (61.9%)
Fibromyalgia	13 (61.9%)
Irritable bowel syndrome (IBS)^d^	8 (38.1%)
Health-related quality of life: mean (SD)	
Physical health (aggregate score) (SF-36)	34.4 (7.5)
Physical component summary (SF-36 PCS)	35.5 (8.6)
Mental component summary (SF-36 MCS)	30.2 (13.5)

^a^At assessment, ^b^some patients have more than one comorbidity, ^c^post-hoc analysis from SCAN interview, ^d^using Rome IV criteria. However, the SCAN interview does not include the item ‘related to defecation’, hence percentage with IBS is likely underestimated

At assessment, the most prevalent patient rated predominant symptoms were abdominal pain (19.1%), tension type headache (19.1%) and backache (14.3%). The patients were moderately to severely impaired due to symptoms, e.g. high degree of school absence or dropped out of school, withdrawal from friends or stopped participating in previous leisure activities. Complete baseline characteristics are presented in Table 2.

### Feasibility

Feasibility evaluation pertained to the group intervention and included treatment adherence and satisfaction and modifications of treatment content and format based on evaluations.

#### Adherence to group treatment

A total of three groups received treatment with 7, 8 and 6 patients in groups 1, 2 and 3, respectively. Two patients dropped out of therapy after the first module, one from pilot group 1 and one from pilot group 2. Both patients were ambivalent about the group format before starting therapy. Of the remaining 19 patients, the overall attendance rate was 93% with 9 patients attending all modules (i.e. 9 modules), 9 patients missing one module and 1 patient missing 3 modules.

#### Satisfaction with group treatment

**Fig. 3:**
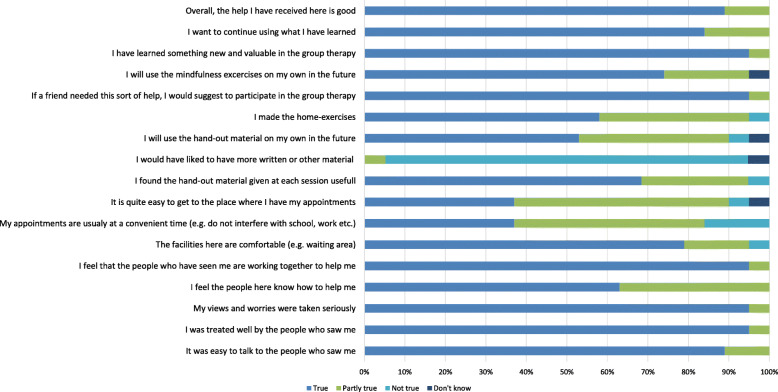
Experience of service questionnaire – adolescents

Apart from the need for more handout material, the adolescents had positive responses regarding the group treatment (Fig. 3). An open-end question regarding the experience of attending group therapy gave rise to several comments addressing the positive fellowship of the group setting (e.g. “it was really good to meet and talk to others who know the daily life with BDS”, “the fellowship of the group was really good” and “we worked well together in the group which made the atmosphere relaxed – so I felt safe”.

#### Feedback close relatives

**Fig. 4:**
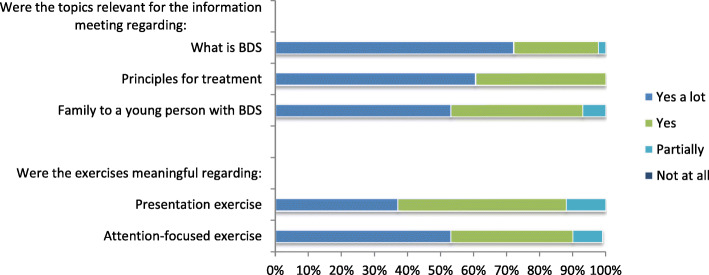
Feedback from information meeting for close relatives (*n* = 43). *Presentation exercise*: An introduction exercise where one family pairs up with another family. From a scripted set of questions, they interview each other and afterwards present the other family to the whole group of close relatives. The presentation exercise is similar to what the adolescents do in the first module. *Attention-focused exercise*: A short mindfulness exercise that resembles what is being done during the adolescents’ treatment

A total of 43 (mainly parents (*n* = 30) but also adolescent siblings (*n* = 4), boyfriends (n = 4) and others (e.g. grandparents, close friends *n* = 5)) filled out the questionnaire distributed at the information meeting for close relatives and provided overall positive feedback regarding the content of the information meeting (Fig. 4). Free text comments included “it was really important to hear the stories from the other families – it made me feel that we are not alone”, “it’s important to be involved in the process” and “great balance between knowledge, dialogue, exercises and theory”.

In the third pilot group, the visit from a former patient and a close relative was evaluated. Out of 22 answers, 77% rated this as very valuable, 9% rated it valuable and 14% did not answer the question. Several free text comments were made regarding this for example “an extremely important meeting and uplifting with a real-life example” and “the visit from a former patient awakens a long-lost hope. Seven years of struggle has been replaced by a hope for a better life for my daughter and our family”.

Ninety-eight percent thought it would be relevant to offer more sessions for close relatives during the group treatment of the adolescents, and 70% could be interested in a discussion group for close relatives.

#### Modifications to group treatment

Based on the patient feedback and the therapists’ experience with the program, the following ad hoc modifications were made to the group treatment: 1) More experience-based exercises (added after pilot group 1), 2) cutback of overall content including written material and a slide presentation with further psychoeducation (after pilot group 1), 3) recorded audio files with therapist voice-guided mindfulness-based exercises for home training (added in pilot group 1), 4) addition of a 10th module/follow-up meeting not originally planned but requested by patients in pilot group 1 (added in pilot group 1), and 5) the introduction of a visit from a former patient and parent (added in pilot group 3).

### Preliminary outcome

Evaluation of potential efficacy was primarily explored for the whole intervention including assessment, psychoeducation and group treatment but separate results for change from group-start to follow up were also explored.

#### Primary outcome

**Fig. 5:**
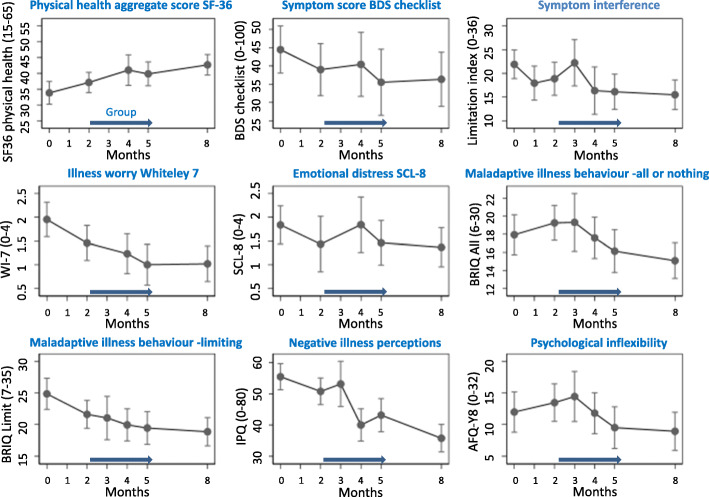
Results. Time point 0: Baseline (assessment), 1: Psychiatric consultation, 2: Before therapy, 3: after 4th module, 4: after 8th module, 5: after 9th module i.e. End of Treatment (EOT), 8: 3-month follow-up (FU)

Patients reported a clinically relevant improvement in self-perceived physical health (SF-36) from assessment to Follow-up (FU, i.e. 3 months after end of treatment). The mean score changed from 33.8 to 42.7 with an overall change of 8.9 (95% CI [5.4; 12.4]; SRM 0.91, 95% CI [0.26;1.57]) (Table 3, Fig. 5). From group start to FU, the change was 5.5 (95% CI [2.8; 8.2]; SRM 0.94, 95% CI [0.40;1.49]). The norm data for Danish women aged 16–24 years show a mean score of 54.

**Table 3:** Mean, confidence intervals and change in outcome measures

Month	0	0,5	2	3	4	5	8	Change from baseline to FU (CI)	SRM From baseline to FU	Change from group start to FU (CI)	SRM from group start to FU
Outcome measure	Assessment Baseline	PC	Group start	Module 4	Module 8	Module 9 End of treatment	Follow up meeting 3-month FU				
	Mean (CI)	Mean (CI)	Mean (CI)	Mean (CI)	Mean (CI)	Mean (CI)	Mean (CI)				
Primary outcome											
SF-36, PPH n = 6–20	33.8 (30.2;37.4)	X	37.1 (33.9;40.3)	X	41.0 (36.2;45.9)	39.9 (36.1;43.6)	42.7 (39.4;46.0)	8.9 (5.4;12.4)	0.91 (0.26;1.57)	5.6 (2.5;8.7)	0.94 (0.40;1.49)
Secondary outcomes											
BDS checklist n = 6–21	44.5 (38.0;50.9)	X	39.0 (31.9;46.1)	X	40.4 (31.7;49.2)	35.5 (26.5;44.6)	36.3 (28.9;43.8)	−8.1 (−14.2;-2.0)	0.52 (− 0.08;1.13)	−2.7 (−9.7;4.4)	0.62 (− 0.26;1.51)
LI n = 6–21	21.9 (18.9;24.9)	17.9 (14.3;21.5)	18.9 (15.4;22.4)	22.2 (17.3;27.1)	16.4 (11.4;21.3)	16.1 (12.4;19.8)	15.5 (12.4;18.6)	−6.4 (−9.7;-3.2)	0.72 (0.21;1.23)	−3.4 (−7.1;0.3)	0.33 (− 0.23;0.89)
WI-7 *n* = 12–21	2.0 (1.6;2.3)	X	1.5 (1.1;1.8)	X	1.2 (0.8;1.7)	1.0 (0.6;1.4)	1.0 (0.6;1.4)	−0.9 (−1.3;-0.6)	0.82 (0.30;1.34)	−0.4 (− 0.8;-0.1)	0.78 (0.26;1.30)
SCL-8 n = 6–21	1.8 (1.4;2.2)	X	1.4 (0.8;2.0)	X	1.8 (1.3;2.4)	1.5 (1.0;1.9)	1.4 (1.0;1.8)	−0.5 (− 0.8;− 0.1)	0.49 (0.02;0.97)	-0.1 (− 0.6;0.5)	0.09 (− 0.71;0.89)
Treatment targets											
BRIQ, an n = 6–20	17.9 (15.7;20.2)	X	19.3 (17.3;21.2)	19.3 (16.1;22.5)	17.6 (15.3;19.9)	16.1 (13.8;18.5)	15.1 (13.1;17.0)	−2.9 (−5.2;-0.4)	0.38 (−0.19;0.94)	−4.2 (−6.3;-2.0)	0.72 (0.21;1.22)
BRIQ, li n = 6–20	24.8 (22.4;27.3)	X	21.6 (19.4;23.8)	21.0 (17.6;24.4)	19.9 (17.4;22.5)	19.5 (16.9;22.0)	18.9 (16.6;21.1)	−6.0 (−8.4;-3.5)	1.00 (0.32;1.67)	−2.7 (−4.9;-0.6)	0.51 (0.03;0.99)
IPQ n = 6–21	55.5 (51.3;59.7)	X	50.8 (46.5;55.1)	53.2 (45.9;60.5)	40.0 (34.9;45.2)	43.1 (37.8;48.5)	35.8 (31.4;40.1)	−19.7 (−24.8;-14.6)	1.22 (0.62;1.82)	−15.0 (− 20.1;-9.9)	0.99 (0.44;1.55)
AFQ-Y8 n = 6–20	12.0 (8.8;15.1)	X	13.5 (10.5;16.4)	14.4 (10.5;18.4)	11.8 (8.6;15.0)	9.5 (6.2;12.8)	8.9 (5.9;11.9)	−3.0 (−5.4;-0.6)	0.57 (−0.02;1.16)	−4.5 (−6.6;-2.4)	0.99 (0.43;1.54)

Mean at different time points as well as change from baseline to follow-up and from group-start to follow up with inclusion of effect sizes

*Abbreviations* alphabetically presented: *AFQ-Y8* avoidance and fusion questionnaire, *BDS* bodily distress syndrome, *BRIQ, AN* behavioural response to illness questionnaire, all or nothing behaviour, *BRIQ, LI* behavioural response to illness questionnaire, limiting behaviour, *CI: 95%* confidence interval, *FU* follow up, *IPQ* illness perception questionnaire, *LI* limitation index, *MCS* mental component summary, *PC* psychiatric consultation, *PCS* physical component summary, *PPH* perceived physical health, *SCL-8* symptom checklist, *SE* standard error, *SRM* standardized response mean, *WI-7* whiteley-7, *X* no measurement

#### Secondary outcomes

Improvement was seen on all secondary outcome measures, including symptom burden (BDS checklist), limitation due to symptoms (LI), illness worry (WI7) and emotional distress (SCL-8) from assessment to FU (Table 3, Fig. 5). When evaluating the change from start of group therapy, further improvement after clinical assessment and initial psychoeducation was only seen for illness worry and symptom burden.

At FU 13 out of 19 adolescents (68.4%) rated that the treatment had shown an overall noticeable positive difference (Table 4).

**Table 4:** Patients’ global impression of change at 3 months after end of treatment (*n* = 19)

No change	- or condition has got worse	0
Almost the same	- hardly any change at all	1
A little better	- but no noticeable change	2
Somewhat better	- but the change has not made any real difference	3
Moderately better	- and a slight but noticeable change	5
Better	- and a definite improvement that has made a real and worthwhile difference	5
A great deal better	- and a considerable improvement that has made all the difference	3

#### Treatment targets

The specific illness-related behaviours ‘all or nothing behaviour’ (− 2.9, 95% CI [− 5.2;-0.4]) and ‘limiting behaviour’ (− 6.0, 95% CI [− 8.4;-3.5]) both showed a decline from assessment to FU with the largest change in limiting behaviour. The change was also seen from group start to FU (Table 3). Furthermore, a decline was seen in the patients’ maladaptive illness perception from both assessment to FU and from group start to FU.

A reduction in two components of psychological inflexibility (i.e. avoidance and fusion) was seen from both assessment (− 3.0 95% CI [− 5.4;-0.6]) and group start (− 4.5 95% CI [− 6.6;-2.4]) to FU. The total score reflecting psychological inflexibility at FU (8.9 95% CI [5.9;11.9]) was similar to the level reported by healthy school age girls, grade 5–10 (*n* = 685) 9.2 (SD 6.4) [54].

## Discussion

### Principal findings

The main finding of this pilot study was that AHEAD was a feasible intervention in regard to treatment satisfaction and adherence to treatment. The adolescents had a low dropout rate of only 10%, which was considerably lower than the dropout rates of 29 and 21% respectively reported in meta-analyses of CBT and ACT interventions for children and adolescents in a range of conditions [81, 82]. Moreover, we found clinically relevant improvements in physical health (primary outcome) as well as significant improvements on most secondary outcomes. The findings are in accordance with the previously reported positive impact of psychological treatment on symptom load and functioning in children and adolescents with FSS [24–26, 28] and of ACT-based interventions in adults with FSS [29–31]. However, it can be questioned whether the positive changes seen in this uncontrolled study can be explained solely by the thorough assessment and psychoeducation, as seen in an RCT in youth with CFS [83]. The importance of a thorough assessment and psychoeducation has been highlighted as an essential first step in management of FSS both in adults and youth [8, 84, 85]. In the present study further improvement was however seen from group-start to follow up on most outcome measures and whether this is solely due to assessment and psychoeducation or also influenced by the group treatment warrants testing in a randomised trial.

Likewise, we cannot rule out that the positive changes can be explained by natural illness course or regression to the mean. However, we regard spontaneous remission to be rather unlikely due to the chronicity of the illness among the participants with a mean illness duration of 4.25 years. Importantly, improvement in physical health was consistent with positive changes in adaptive illness perceptions and behaviours. Hence future research should assess whether these changes are central for treatment outcome in adolescents with FSS as found in adults with a range of FSS [17, 86–88].

### The generic group-based treatment approach

Our pilot study report on one of the first generic treatments for adolescents presenting with multiple FSS, and the data on the patients’ and relatives’ satisfaction with the group-treatment, low dropout rate and positive outcomes indicate the feasibility of this approach. These findings are supported by another pilot study on Mindfulness Based Stress Reduction (MBSR) for adolescents with a range of FSS, concluding that it was a feasible and acceptable intervention [42]. In adults with FSS, it has been discussed whether the development of specifically tailored treatments for each FSS or symptom profile can be an inefficient strategy due to the costly nature of establishing separate clinics in each medical (sub)specialty, the fragmented care available and difficulty in handling multisymptomatic patients at those clinics [43–45, 84, 89]. Furthermore, the experience from the present trial could suggest that treatment of youth with multiple FSS requires skilled therapists who have received specific training in the interventions used (e.g. CBT or ACT) but also have extensive knowledge on FSS and clinical experience with regard to treatment of this patient group. Based on our experience, a specific issue for the non-medically trained professionals including psychologists, may be the uncertainty on how to deal with the frequent (and sometimes worrying or even alarming) physical symptoms complaints the patients present with. In the present study we therefore chose to have pairs of therapists (one physician, one psychologist) to deliver the group therapy. More research is required regarding the training and clinical experience needed to treat this group of patients with AHEAD or similar interventions with potential focus on the need for supervision or support regarding medical issues of non-medical trained professionals. Thus, despite that our findings are preliminary and based on a limited number of patients, offering a generic program for different types of FSS in adolescents could be an important first step towards more accessible treatments for the patient group. Hence further research including RCTs is warranted.

When choosing an ACT-based approach for our intervention, an implied assumption was a higher degree of psychological inflexibility in the patient group. The measure (i.e. AFQ-Y8) from assessment suggest that our patient group did have a higher level of avoidance and fusion as compared to general high school students [78], supporting a rationale for an ACT-based intervention. From pre- to follow-up assessment (i.e. after group-start), there was a decrease in AFQ-score. Although tentative, this indicates that the group treatment reduced the levels of experiential avoidance and cognitive fusion. This is promising given the previously found relationship to pain and disability in youths, and should be further evaluated in future clinical trials [90].

The involvement of caregivers is often seen as a key component when treating children and adolescents [91]. In children and adolescents with FSS, this is supported by the findings of several studies, where parents inadvertently may play a role in reinforcement of maladaptive illness behaviours and beliefs [19, 20, 92, 93]. In our pilot study, close relatives were involved in the assessment, the following psychiatric consultation and in the information meeting, and they were strongly encouraged to participate in the individual consultation after 8th module. The relatives’ participation was considered an important aspect of the treatment; however, the intended focal point was the group effect within the adolescent group. Hence, despite the relatives’ wish for more involvement (e.g. more sessions for close relatives), the program was not modified accordingly. Recent treatment studies have specifically targeted parents of children with chronic pain and shown improved parental psychological flexibility intending to diminish parental distress and promote more adaptive responses to their child’s pain [94, 95]. However, in a different patient group (i.e. young patients with anxiety) several meta-analyses show that CBT programs with and without active parental involvement show comparable efficacy at post-treatment; thus not a clear superiority when involving parents more actively [96, 97]. Further empirical support is therefore pivotal in deciding on how and how much to involve relatives in treatment to make the most of their potential positive impact. An important future focus point is also the implications of age and maturity level on feasibility and treatment effect including the potential shift in primary relatives from parents to others when the adolescents approach adulthood.

### Strengths and limitations

Our findings should be interpreted in the light of some limitations. First, the uncontrolled study design with a small sample size limits the validity of the overall conclusions from the study. The choice of design was based on the main focus of the study, i.e. the feasibility evaluation of the new group-based ACT intervention which was not limited by the sample size. The observed relevant improvements on several outcomes after assessment and during treatment with AHEAD should be replicated in a larger RCT to establish clinical relevance. Second, not all questionnaires were distributed in all three pilot groups at the same time-points (see Additional file 3) due to considerations regarding respondence burden which may influence the validity of the study. Third, more clear a priori criteria for assessing success of feasibility (e.g. registration of additional contact with close relatives, more specific evaluation of recruitment rates) could potentially have influenced the interpretation of the results (e.g. indications of whether the study population presented a highly motivated group of patients and close relatives). However, it was the clinical experience that close relatives very seldom contacted the therapists outside of the scheduled meetings (i.e. information meeting for close relatives and the individual consultation). Fourth, the ad hoc changes made to the AHEAD program during the study may limit the validity of the evaluation of the overall treatment. However, the overall themes and content remained the same with only smaller mainly contextual changes (e.g. removing power-point presentation). Fifth, the inclusion of only girls, and the fact that only two clinicians provided all treatment, reduces the generalisability of the results. Furthermore, assessment of treatment fidelity, registration of potential adverse events and other treatment received, as well as incorporation of specification for the training of therapists are important aspects to include in upcoming trials.

Strengths of the present study include all patients undergoing a thorough systematic psychiatric and somatic assessment to ensure absence of underlying well-defined somatic or psychiatric disorders as primary cause of symptoms. Furthermore, well-defined in- and exclusion criteria were applied, and validated outcome measures were employed. Patients with comorbidity of anxiety and depression were included, which increases the generalisability as these are common in youth with FSS [3–5]. Finally, the group-treatment was manualised with a detailed description of each treatment module (both patient and therapist version).

### Clinical implications

FSS in children and adolescents are associated with increased societal costs including extensive health care visits [98, 99], school absence and parental absence from work [100]. Considering the suggested clinically relevant improvements in self-reported physical health and the high treatment satisfaction rated by the adolescents and parents and as observed in a good adherence rate, AHEAD may have the potential to improve quality of life and reduce illness-related impairment. Given the risk of continuity of symptoms into adulthood, AHEAD also holds the potential to reduce life years lived with disability (YLDs) and thus potentially decrease societal costs.

## Conclusion

Group-based ACT added to a comprehensive clinical assessment was a feasible and potentially efficacious treatment for adolescents with multiple FSS. The results from the present study warrant testing of the efficacy of AHEAD in a larger randomised controlled trial.

## Supplementary information

**Additional file 1.**

**Additional file 2.**

**Additional file 3.** Overview of distribution of questionnaires to specific pilot groups.

**Additional file 4.** Questions used for evaluation of information meeting for close relatives.

## Data Availability

The data that support the findings of this study are available on request from the corresponding author Karen Hansen Kallesøe. The data are not publicly available due to them containing information that could compromise research participant privacy and consent.

## Abbreviations

ACT: Acceptance and commitment therapy
AFQ: Avoidance and fusion questionnaire
AHEAD: Acceptance and commitment therapy for health in adolescents
BDS: Bodily distress syndrome
BRIQ: Behavioural response to illness questionnaire
CBT: Cognitive behavioural therapy
CI: Confidence interval
EOT: End of treatment
FSS: Functional somatic syndromes
FU: Follow up
IPQ: Illness perception questionnaire
LI: Limitation index
MCS: Mental component summary
PCS: Physical component summary
RCT: Randomised controlled trial
SCL: Symptoms checklist revised
SD: Standard deviation
SRM: Standardized response mean
