# Supplementary material for: Feasibility of group-based acceptance and commitment therapy for adolescents (AHEAD) with multiple functional somatic syndromes: a pilot study
Source: BMC Psychiatry. 2020 Sep 21;20:457. doi: 10.1186/s12888-020-02862-z (PMC7507241; doi:10.1186/s12888-020-02862-z)
Supplement: Supplementary file 1 — Additional file 1. [file 12888_2020_2862_MOESM1_ESM.docx]

**Appendix 1**

Material used in the assessment and for the initial psychoeducation

**Assessment: The use of a chronological overview**

As part of the assessment the young person’s contacts with the health care system (all examinations, results and treatments from hospitals and medical specialists) were registered on the left side of a chronological overview written on a blackboard. On the other side of this overview all important events (both positive and challenging) in the adolescent’s social life were registered (Figure A). It provided a helpful overview of life-stressors, health care contacts and previous treatments and often the patients and parents realized a potential connection between life-stressors and symptom development.


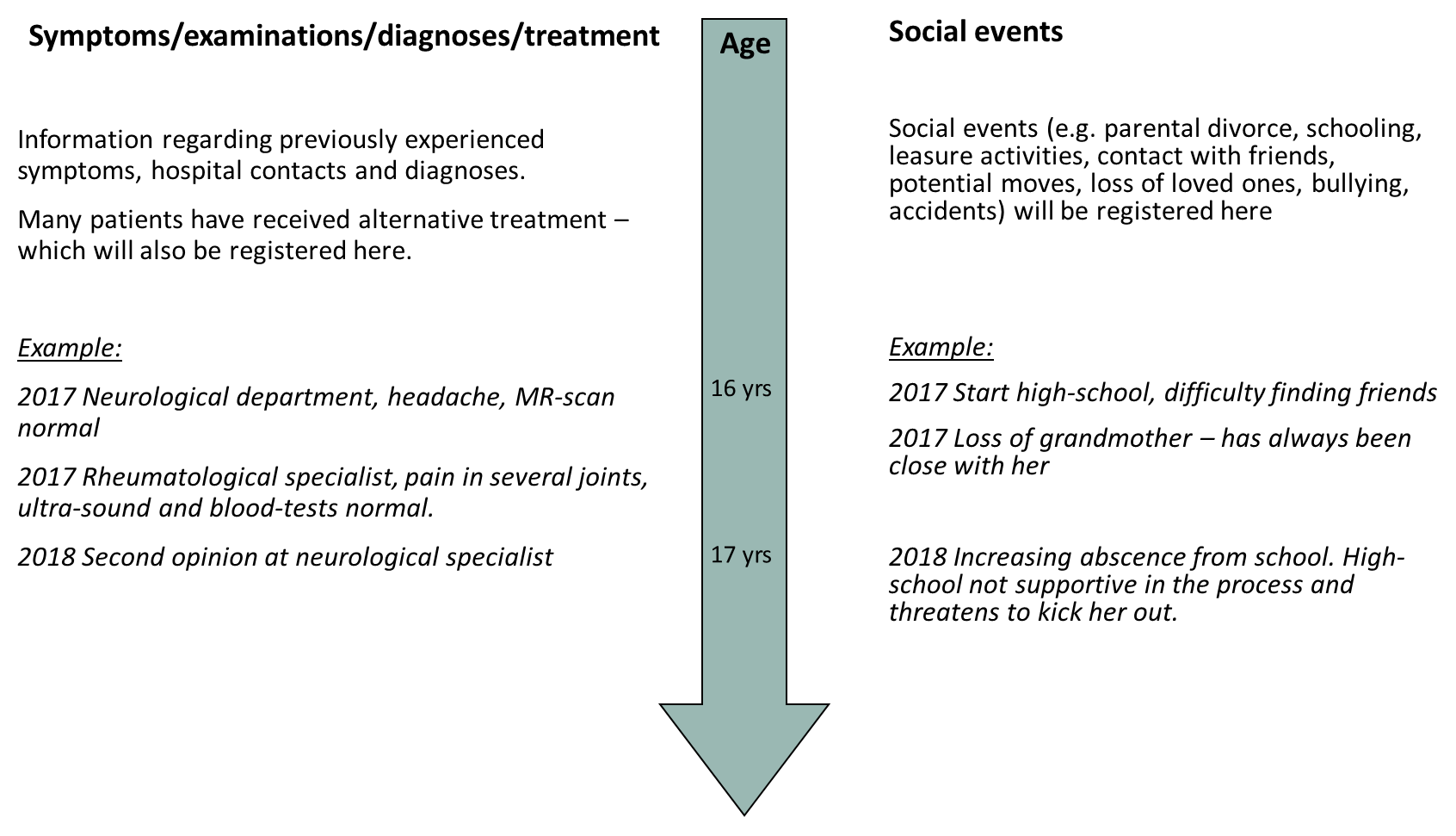


**Figure A. Chronological overview**

**Material for the psychoeducation immediately following the assessment**

**General explanatory biopsychosocial model for symptoms**

During assessment and especially in the making of the chronological overview (Figure A), most patients and parents disclosed a range of potentially predisposing, precipitating and perpetuating factors of relevance in the overall understanding of symptoms in a bio-psycho-social context. After assessment, a general explanatory model for symptom development and perpetuation (Figure B) was presented to the adolescent and the parents with incorporation of the specific relevant individual factors drawn on a blackboard. The detailed incorporation of individual factors most often facilitated a broader understanding of symptom development and potential important perpetuating factors that could be addressed in further treatment.


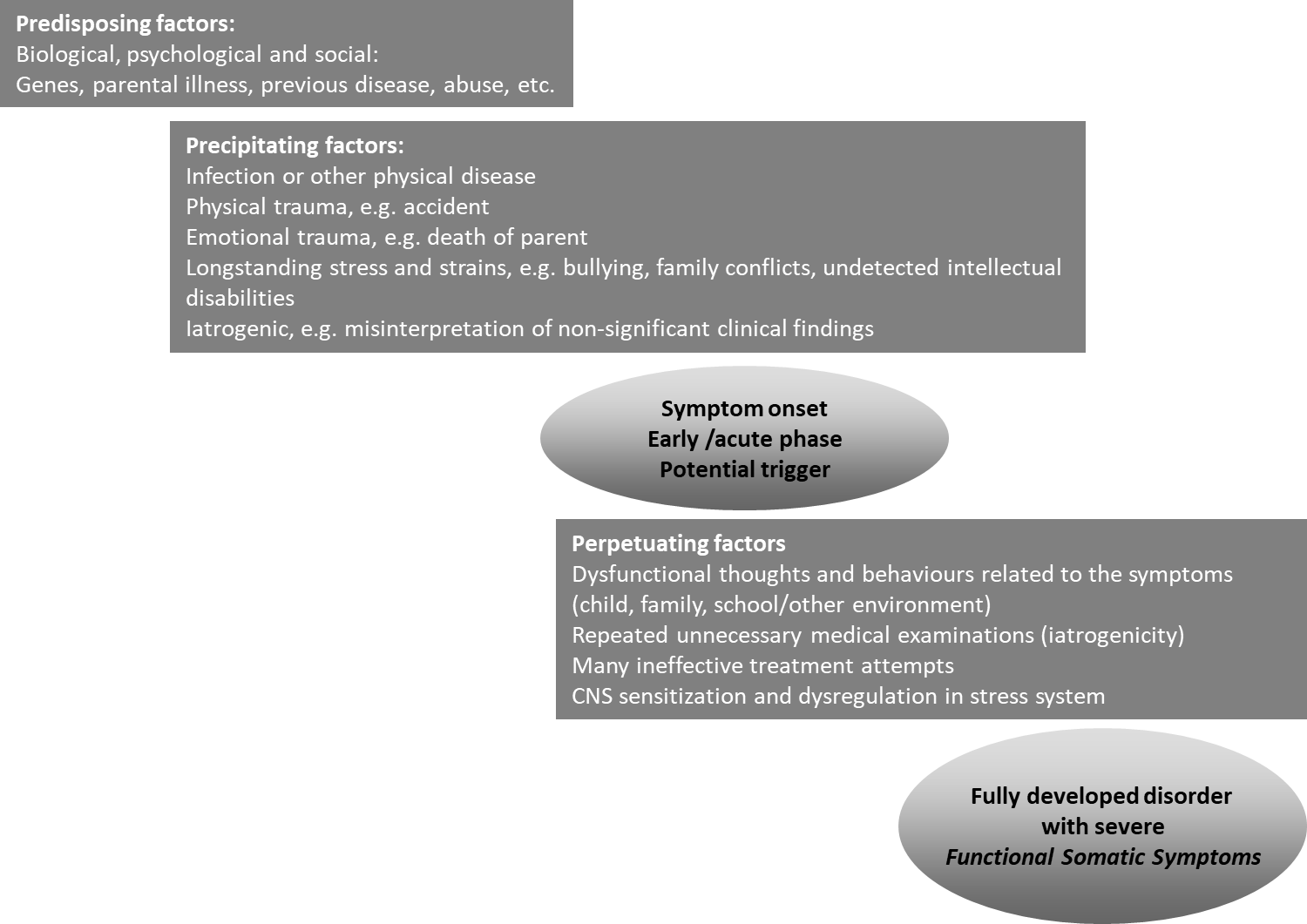


**Figure B. General explanatory biopsychosocial model for symptoms** as proposed by Rask et al. (1)

**Common illness-related behaviour – ”All-or-nothing”**

Patients with BDS often develop an illness-related behaviour called “all-or-nothing” behaviour (Figure C) which is known to perpetuate FSS (2). The behaviour is characterised by an activity pattern alternating between periods of high activity when symptom levels are low followed by prolonged periods of rest with low level of functioning secondary to increased symptom levels. During psychoeducation following assessment, the patients were introduced to the pattern and asked if they could recognize it. Furthermore, they were informed of a strategy to break the pattern i.e. to take gradual steps from a low starting point towards a higher level of functioning (Figure D).

**Figure C. Development of BDS and maladaptive “all-or-nothing” behaviour**


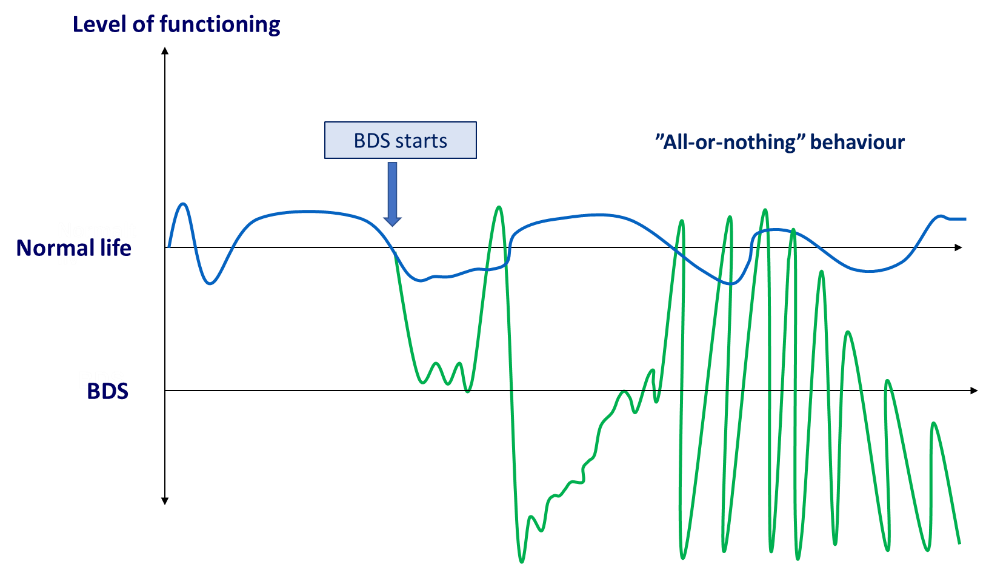


**Figure D. Alternative adaptive behaviour – gradual rehabilitation**


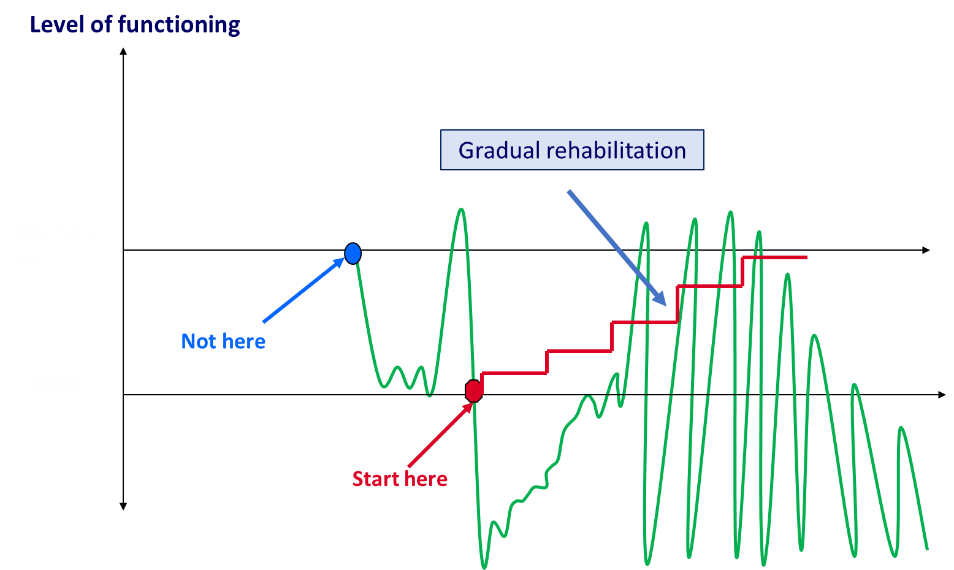


**Further symptom explanation based on "the filter model"**

After assessment and the provision of the general explanatory model with a bio-psycho-social focus, we also provided a simpler explanation for symptom development focusing on potential involved (neuro)biological factors based on neuro-psycho-behavioural theories) (3).

We introduced that symptoms can arise from:

- Increased symptom-production (arousal – “stress”)
- Increased symptom perception – “defect filter”

The filter model (Figure E) was presented and explained in line with the below description.

“Research suggests that patients with BDS have an increased sensibility towards bodily signals. The body sends signals to the brain constantly. Healthy individuals have a filter that stops a lot of the signals before they reach the brain meaning that they do not register these signals as the filter only lets the most important signals get through. Research shows that this filter is malfunctioning in individuals with BDS. The filter has "holes" that let more signals get through to the brain. This could explain that you can have a normal scan of for instance the back, but still feel pain.” (From the general psychoeducation material from the Research Clinic for Functional Disorders and Psychosomatics (4)).


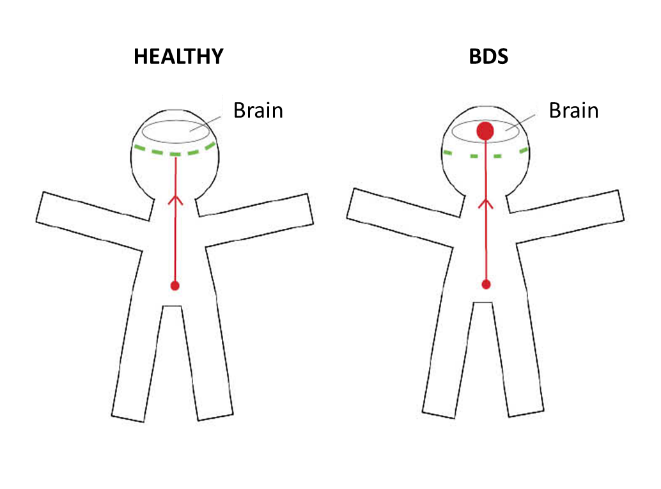
 **Figure E. The filter model (4)**

References

1. Rask CU, Bonvanie IJ, Garralda ME. Risk and Protective Factors and Course of Functional Somatic Symptoms in Young People. In: Hodes M, Gau S, Petrus De Vries SG, editors. Understanding Uniqueness and Diversity in Child and Adolescent Mental Health 1st ed: Academic Press; 2018. p. 77.

2. Sullivan N, Phillips LA, Pigeon WR, Quigley KS, Graff F, Litke DR, et al. Coping with Medically Unexplained Physical Symptoms: the Role of Illness Beliefs and Behaviors. International journal of behavioral medicine. 2019;26(6):665-72.

3. Henningsen P, Gündel H, Kop WJ, Löwe B, Martin A, Rief W, et al. Persistent Physical Symptoms as Perceptual Dysregulation: A Neuropsychobehavioral Model and Its Clinical Implications. Psychosom Med. 2018;80(5):422-31.

4. Bodily Distress Syndrome (BDS). The Research Clinic for Functional Disorders AUH, Denmark, 2012.https://funktionellelidelser.dk/fileadmin/www.funktionellelidelser.au.dk/patient_Pjecer/Patientinformation__BDS_.pdf
