# Supplementary material for: Feasibility of group-based acceptance and commitment therapy for adolescents (AHEAD) with multiple functional somatic syndromes: a pilot study
Source: BMC Psychiatry. 2020 Sep 21;20:457. doi: 10.1186/s12888-020-02862-z (PMC7507241; doi:10.1186/s12888-020-02862-z)
Supplement: Supplementary file 2 — Additional file 2. [file 12888_2020_2862_MOESM2_ESM.docx]

Appendix 2. Material for the psychiatric consultation (1½ hrs.)

The psychiatric consultation consisted of the following elements: 1) Additional psychoeducation about multi-organ BDS, 2) Presentation of general health promoting strategies and together with the young going through the stress-resource fraction in detail with an individual choice of two elements to work on (Figure A) and 3) Evaluation of the need for additional help from a school-counsellor, psychologist, physiotherapist, social services, additional psychiatric assessment and/or pharmacological treatment. If needed, the physician would provide further advice for the relevant professionals involved. Furthermore, a written treatment plan was made for the general practitioner.

**
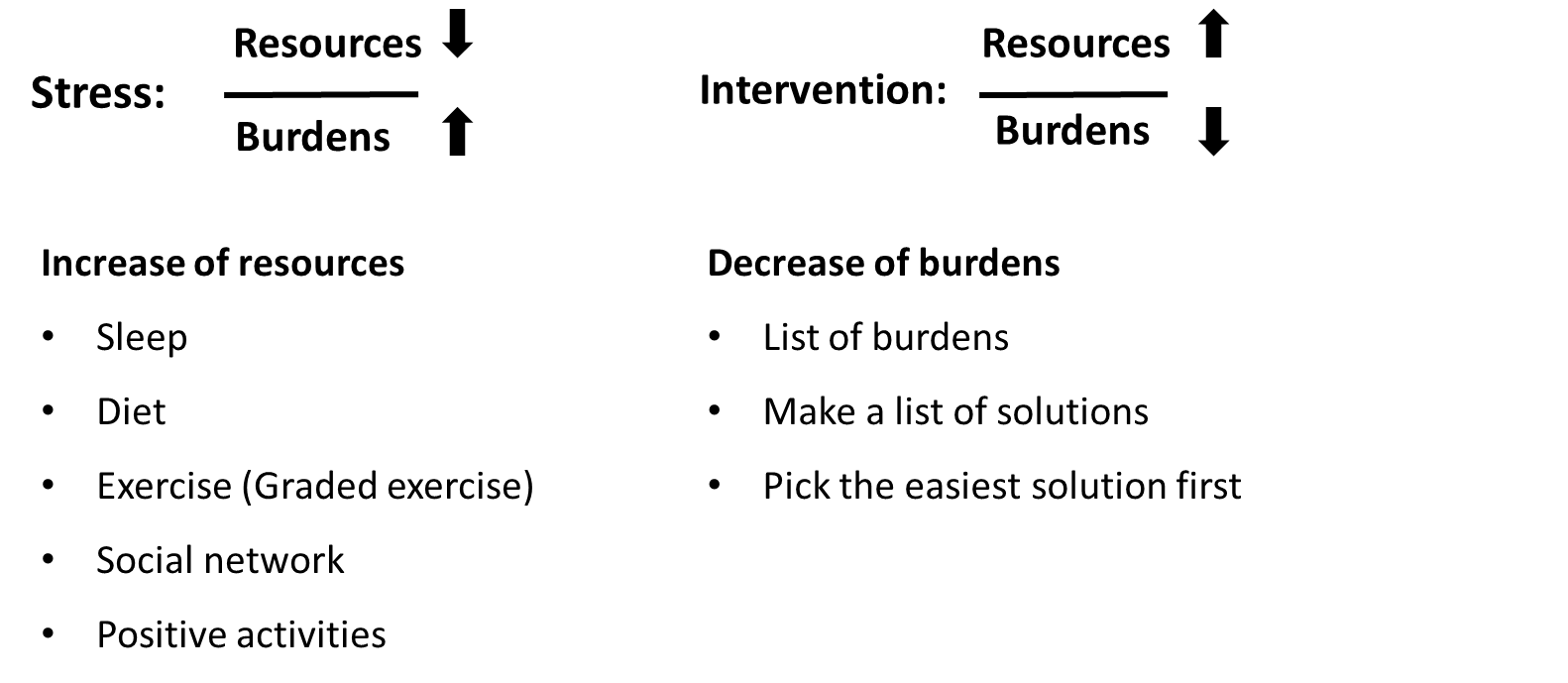
**

**Figure A. Stress-resource fraction**
