# Supplementary material for: Feasibility of group-based acceptance and commitment therapy for adolescents (AHEAD) with multiple functional somatic syndromes: a pilot study
Source: BMC Psychiatry. 2020 Sep 21;20:457. doi: 10.1186/s12888-020-02862-z (PMC7507241; doi:10.1186/s12888-020-02862-z)
Supplement: Supplementary file 3 — Additional file 3. Overview of distribution of questionnaires to specific pilot groups. [file 12888_2020_2862_MOESM3_ESM.doc]

**Appendix 3. Overview of distribution of questionnaires to specific pilot groups**

| **Questionnaire/Timepoint of distribution** | **Baseline ^1^** | **Before psychiatric consultation** | **Before start of group therapy** | **After 4th module** | **After 8th module** | **After 9th module ^2^** | **After 10th module ^3^** |
| --- | --- | --- | --- | --- | --- | --- | --- |
| **SF-36** | 2, 3 |  | 1, 2, 3 |  | 3 | 1, 3 | 1, 2, 3 |
| **Limitation index** | 1, 2, 3 | 1, 2 | 1, 2 | 1 | 3 | 1, 3 | 1, 2, 3 |
| **BDS checklist** | 1, 2, 3 |  | 1, 2 |  | 2 | 1 | 1, 3 |
| **AFQ-Y** | 2, 3 |  | 1, 2, 3 | 1 | 2, 3 | 1, 3 | 1, 2, 3 |
| **PGIC** |  |  |  |  | 3 | 3 | 1, 2, 3 |
| **BRIQ** | 2, 3 |  | 1, 2, 3 | 1 | 2, 3 | 1, 3 | 1, 2, 3 |
| **IPQ** | 1, 2, 3 |  | 1, 2, 3 | 1 | 2, 3 | 1, 3 | 1, 2, 3 |
| **Whiteley-8** | 1, 2, 3 |  | 1, 2, 3 |  | 2, 3 | 1, 3 | 1, 2, 3 |
| **SCL-8** | 1, 2, 3 |  | 1 |  | 3 | 1, 3 | 1, 2, 3 |

Numbers in table correspond to the specific pilot groups i.e. 1= pilot group 1 (6 patients), 2= pilot group 2 (8 patients) and 3= pilot group 3 (7 patients)

^1^ Before clinical assessment

^2^ End of treatment

^3^ Three months after end of treatment
