# Supplementary material for: Feasibility of group-based acceptance and commitment therapy for adolescents (AHEAD) with multiple functional somatic syndromes: a pilot study
Source: BMC Psychiatry. 2020 Sep 21;20:457. doi: 10.1186/s12888-020-02862-z (PMC7507241; doi:10.1186/s12888-020-02862-z)
Supplement: Supplementary file 4 — Additional file 4. Questions used for evaluation of information meeting for close relatives. [file 12888_2020_2862_MOESM4_ESM.docx]

**Appendix 4. Questions used for evaluation of information meeting for close relatives**

| Is the content relevant regarding: | Yes, a lot | Yes | Partly | Not at all |
| --- | --- | --- | --- | --- |
| What is BDS |  |  |  |  |
| Treatment principles |  |  |  |  |
| Being family to an adolescent with BDS |  |  |  |  |
| Former patient with parents share their experiences |  |  |  |  |
| Is the content understandable regarding: | **Yes, easy** | **Yes** | **Partly** | **Not at all** |
| What is BDS |  |  |  |  |
| Treatment principles |  |  |  |  |
| Being family to an adolescent with BDS |  |  |  |  |
| Are the included exercises meaningful regarding: | **Yes, a lot** | **Yes** | **Partly** | **Not at all** |
| Presentation exercise |  |  |  |  |
| Attention focused exercise |  |  |  |  |
| Values-based exercise |  |  |  |  |

Is the timepoint for the meeting ok: Yes No If no, when would it suit you?

Is the duration of the meeting adequate: Yes No If no, how long?

Was there enough time for questions and open discussions? Yes No

Would it be relevant with discussion groups for relatives? Yes No

Would it be relevant to offer additional meetings for relatives during the treatment? Yes No

Other comments?
